# Comparative efficacy and safety of teriparatide versus bisphosphonates in osteoporosis: a meta-analysis

**DOI:** 10.3389/fsurg.2025.1582180

**Published:** 2025-12-08

**Authors:** Huan Jin, Ying Dong, Cai Huang, Di Wang, Ziyi He, Lin Shen, Chen Ma, Zixian Wang, Siwei Shi, Bo Shuai

**Affiliations:** 1 Department of Integrated Traditional Chinese and Western Medicine, Union Hospital, Tongji Medical College, Huazhong University of Science and Technology, Wuhan, China; 2 College of Sports Medicine, Wuhan Sports University, Wuhan, China; 3 Department of Internal Medicine, Rongjun Hostipal of Hubei, Wuhan, China

**Keywords:** teriparatide, bisphosphonates, osteoporosis, vertebral fracture, bone mineral density, meta-analysis

## Abstract

**Objectives:**

This meta-analysis systematically compared the efficacy and safety of teriparatide and bisphosphonates in treating osteoporosis (OP) to inform optimal treatment strategies.

**Methods:**

A comprehensive literature search was conducted in PubMed, Embase, Cochrane Library, Web of Science, and Google Scholar from inception through October 2024. Randomized controlled trials comparing teriparatide and bisphosphonates for OP were included. Primary outcomes included vertebral/non-vertebral fracture risk reduction and lumbar spine/femoral neck bone mineral density (BMD) improvements. Data were analyzed using RevMan 5.3, with dichotomous data assessed via odds ratios (OR) and continuous data via mean differences (MD), both reported with 95% confidence intervals (CI).

**Results:**

Thirteen studies involving 4,420 patients were analyzed (six Grade A, four Grade B, three Grade C). Teriparatide significantly reduced vertebral [OR = 0.40, 95% CI = (0.33, 0.49), *p* < 0.00001] and non-vertebral fracture risks [OR = 0.58, 95% CI = (0.54, 0.70), *p* < 0.00001] compared to bisphosphonates. At 12 months, teriparatide showed greater improvements in lumbar spine BMD [MD = 2.72, 95% CI = (2.44, 3.00), *p* < 0.00001] and femoral neck BMD [MD = 1.66, 95% CI = (0.42, 2.90), *p* = 0.0090]. In studies with >18-month follow-ups, teriparatide maintained superior lumbar spine BMD [MD = 4.65, 95% CI = (4.28, 5.03), *p* < 0.00001] and femoral neck BMD [MD = 1.42, 95% CI = (0.57, 2.26), *p* = 0.0010] improvements. Adverse event rates were comparable between teriparatide and bisphosphonates [MD = 1.03, 95% CI = (0.88, 1.20), *p* = 0.73].

**Conclusion:**

Teriparatide demonstrated superior efficacy in reducing vertebral/non-vertebral fracture risks and improving BMD in both short- and long-term treatments, with a safety profile comparable to bisphosphonates.

## 1 Introduction

Osteoporosis (OP) is a prevalent bone disease characterized by decreased bone mass and structural deterioration, which increases bone fragility and susceptibility to fractures (1). Treatment of OP re-quires a balanced approach that reduces bone resorption and pro-motes bone formation. Although treatment strategies have increasingly shifted toward fracture risk-based approaches, with teriparatide being recommended as a first-line therapy for patients at very high fracture risk, bisphosphonates remain the mainstream treatment option for most OP cases (2).

Teriparatide, consisting of the N-terminal 34 amino acids of par-athyroid hormone, mimics the effects of parathyroid hormone by activating osteoblasts, thereby accelerating bone formation, increasing bone density, and reducing fracture risk (3). Moreover, common bisphosphonates, such as alendronate, risedronate, and zoledronic acid, are considered the primary treatment choices for various types of OP owing to their cost-effectiveness, proven efficacy, and long-term safety (4). Both teriparatide and bisphosphonates effectively improve bone density and mitigate fracture risk in patients with OP (5). However, owing to their distinct mechanisms of action, differences in efficacy and safety profiles exist. Although substantial research has demonstrated the effectiveness and safety of teriparatide and bisphosphonates in OP treatment. At the same time, previous randomized controlled trials, including the VERO trial (6), have demonstrated that teriparatide significantly reduces the risk of new vertebral and non-vertebral fractures compared to bisphosphonates. For patients with high fracture risk, teriparatide may be a more effective treatment option. Although the superiority of teriparatide over bisphosphonates has been demonstrated in previous studies, several critical gaps remain. Earlier meta-analyses included limited recent data, lacked subgroup analyses by population region or other subgroup, and seldom addressed the occurrence of serious adverse events. Therefore, this updated meta-analysis aimed to incorporate newly available randomized controlled trials, explore potential sources of heterogeneity through subgroup and sensitivity analyses, and provide an expanded assessment of long-term efficacy and safety to refine the clinical applicability of teriparatide therapy.

## 2 Materials and methods

### 2.1 Literature search

**Figure 1:**
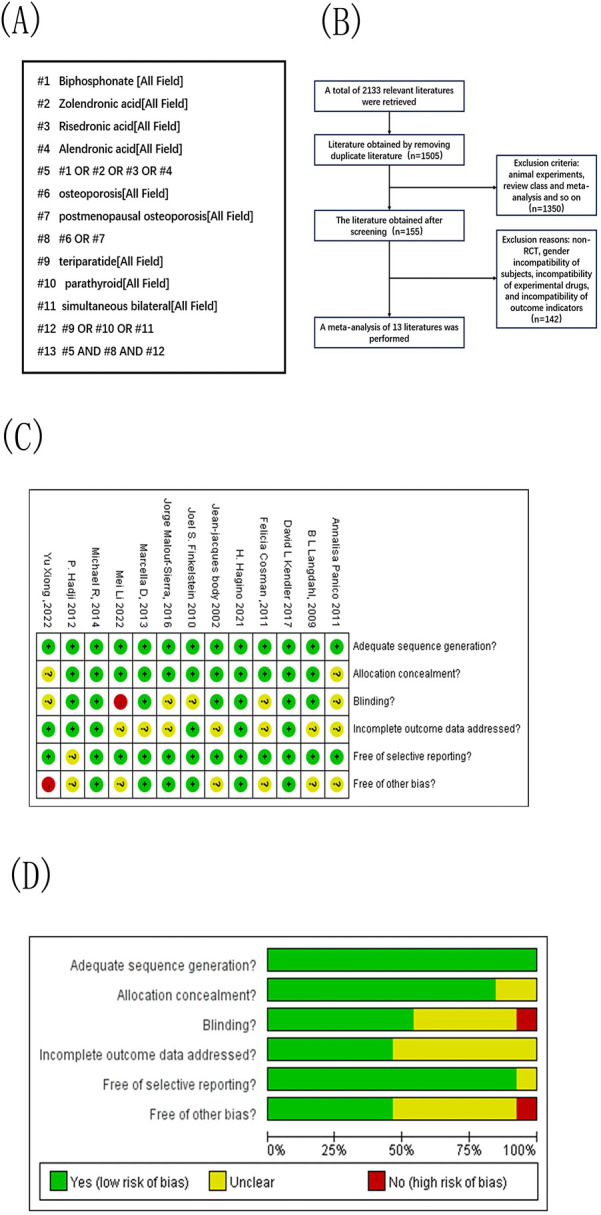
Document extraction and risk assessment map. **(A)** Literature retrieval strategy; **(B)** Literature screening flow chart; **(C)** Integrated migration map for quality assessment of included studies, “+” low-risk, “? “ Unknown-risk, “-” high-risk; **(D)** Included study quality assessment bias risk bar chat;.

Systematic reviews and meta-analyses carried out in this study strictly adhered to the Preferred Reporting Items for Systematic Reviews and Meta-Analyses (PRISMA) guidelines. A comprehensive literature search was conducted using PubMed, Embase, Cochrane Library, Web of Science, and Google Scholar. As an example, the search strategy used in PubMed is outlined in Figure 1A. The primary outcomes included reductions in vertebral and non-vertebral fracture risks and improvements in the lumbar spine and femoral neck bone mineral density (BMD).

### 2.2 Inclusion and exclusion criteria

The inclusion criteria were (1) Study type: Randomized controlled trials (RCTs) comparing the efficacy of teriparatide and bisphosphonates in treating OP, with calcium and vitamin D as baseline medications; (2) Study participants: Patients aged 45–85 years diagnosed with OP (eligible for inclusion if they met the diagnostic criteria for OP).; (3) Outcome measures: Incidence of >1 vertebral or non-vertebral fracture, changes in vertebral and femoral neck BMD over different durations, and assessment of adverse events.

The exclusion criteria were (1) Studies with additional interventions beyond teriparatide or bisphosphonates; (2) Animal studies or review articles; (3) Studies lacking detailed data, with duplicate data, or not aligned with the research objectives.

### 2.3 Data extraction

Two researchers with relevant expertise independently extracted key information based on study objectives and selection criteria, including the first author's name, publication year, sample size, interventions, and outcome measures. Any disagreements were resolved by consulting a third research expert.

### 2.4 Quality assessment

The quality of the included studies was evaluated by two independent reviewers. Any disagreements were resolved by consulting a third research expert. Using the Cochrane risk of bias tool, quality assessment was carried out by evaluating six key areas, namely sequence generation, allocation concealment, blinding, incomplete outcome data, selective outcome reporting, and other sources of bias. Each criterion was scored as “low risk of bias” (1 point), “uncertain risk of bias” (0 points), or “high risk of bias” (-1 point). Studies were categorized into three quality grades, namely, Grade A (5–6 points), Grade B (3–4 points), and Grade C (1–2 points).

### 2.5 Statistical analysis

Meta-analysis was conducted using RevMan 5.3 software (Copenhagen: The Nordic Cochrane Centre, The Cochrane Collaboration, 2014). Dichotomous data were assessed using odds ratios (OR), while continuous data were evaluated using mean differences (MD). All results are presented with their effect sizes and 95% confidence intervals (95% CI). A fixed-effects model was applied if inter-study heterogeneity was low (I^2^ < 50% and *p* > 0.1); else, a random-effects model was employed. Subgroup analysis under a random-effects model was conducted to explore sources of heterogeneity. Sensitivity analysis was performed by sequentially removing individual studies. Publication bias was assessed using a funnel plot. Statistical significance for the meta-analysis was set at *α* = 0.05.

## 3 Results

### 3.1 Literature screening process and results

A total of 2,133 articles were initially identified from English databases. After auto-mated exclusion of non-relevant articles, 1,505 articles remained. Screening titles and abstracts led to the exclusion of 1,350 articles, including animal studies, reviews, and meta-analyses, resulting in 155 articles for full-text review. Based on eligibility criteria concerning participants, interventions, and outcomes, a total 13 articles met the inclusion criteria and were included in the analysis (6–18). A detailed screening flowchart is presented in Figure 1B.

### 3.2 Basic characteristics of included studies

The 13 included studies were all RCTs published between 2002 and 2022, with sample sizes ranging from 20 to 600 participants, totaling 4,420 patients after accounting for dropouts (teriparatide = 2,308; bisphosphonates = 2,112). Among the thirteen studies, seven compared teriparatide with alendronate (7, 8, 13–17), four with risedronate (6, 9, 10, 18), and two with zoledronic acid (11, 12). Detailed study characteristics are listed in Table 1.

**Table 1:** General characteristic of the included studies.

Author	Country	Sex	EG	CG	Sample size (*n*=?)		Age (X ± s)/year		Intervention		Basic treatment/day	Follow-up/month	Outcomes
					EG	CG	EG	CG	EG	CG			
M. Li, 2022	China	F	TPTD	ALN	397	194	64 ± 7	63 ± 6	20 μg/day, SC	70 mg/week, oral	Calcium 500 mg; Vit D 200 IU	12	③④
J. S. Finkelstein, 2010	USA	F	TPTD	ALN	29	20	64 ± 6	65 ± 7	40 μg/day, SC	10 mg/day, oral	Calcium 1,000–1,200 mg; Vit D 400 IU	24	⑤
H. Hagino, 2021	Japan	F	TPTD	ALN	489	496	81 ± 4	81 ± 4	56.5 μg/week, SC	5 mg/day, oral	Calcium 1,000–1,200 mg; Vit D 400 IU	18	①②③④⑤⑥
J. J. Body, 2002	Canada	F	TPTD	ALN	71	72	66 ± 8	65 ± 9	40 ug/day, SC	10 mg/day, oral	Calcium 1,000 mg; Vit D 400–1,200 IU	24	③④⑤⑥
M. R. McClung, 2014	multi-center	F	TPTD	ALN	49	51	67 ± 5	66 ± 5	20 ug/day, SC	70 mg/week, oral	Calcium 1,000 mg; Vit D 800 IU	12	③
B. L. Langdahl, 2009	USA	F	TPTD	ALN	107	106	61 ± 1	62 ± 1	20 ug/day, SC	10 mg/day, oral	Calcium 1,000 mg; Vit D 800 IU	18	③⑤
A. Panico, 2011	Italy	F	TPTD	ALN	42	39	65 ± 9	60 ± 14	20 ug/day, SC	70 mg/week, oral	Calcium 1,000 mg; Vit D 800 IU	18	①⑤⑥
D. L. Kendler, 2017	multi-center	F	TPTD	RID	498	515	65 ± 6	64 ± 2	20 ug/day, SC	35 mg/week, oral	Calcium 500–1,000 mg; Vit D 400–800 IU	24	①②
J. Malouf-Sierra, 2016	UK	F/M	TPTD	RID	86	85	77 ± 8	76 ± 7	20 ug/day, SC	35 mg/week, oral	Calcium 500–1,000 mg; Vit D 800 IU	18	③④⑤⑥
M. D. Walker, 2013	USA	M	TPTD	RID	9	10	54 ± 2	52 ± 4	20 ug/day, SC	35 mg/week, oral	Calcium 500 mg; Vit D 400 IU	18	①②③④⑤⑥
P. Hadji, 2012	multi-center	F	TPTD	RID	360	350	71 ± 9	72 ± 8	20 ug/day, SC	35 mg/week, oral	Calcium 1,000 mg; Vit D 800 IU	18	①②⑤⑥
F. Cosman, 2011	USA	F	TPTD	ZOL	138	137	64 ± 9	66 ± 9	20 ug/day, SC	5 mg/year, IV infusion	Calcium 1,000 mg; Vit D 800 IU	12	①②③④
Y. Xiong, 2022	China	F	TPTD	ZOL	36	41	63 ± 7	64 ± 8	20 ug/day, SC	5 mg/year, IV infusion	Calcium 1,000 mg; Vit D 800 IU	24	③⑥

F, female; M, male; EG, experimental group; CG, control group; TPTD, Teriparatide; ALN, alendronate; RID, risedronate; ZOL, zoledronate; Vit D, vitamin D; Outcomes (the outcomes recorded in the study) ①: Incidence of Vertebral Fractures;②: Incidence of Non-Vertebral Fractures; ③: 12-Month Increase Rate of Lumbar Spine BMD; ④: 12-Month Increase Rate of Femoral Neck BMD; ⑤: 18-Month or Longer Increase Rate of Lumbar Spine BMD; ⑥: 18-Month or Longer Increase Rate of Femoral Neck BMD.

### 3.3 Quality assessment

The overall risk of bias assessment of the included studies is illustrated in Figures 1C,D. Sequence generation was the only item that was rated as low risk across all studies. The risks of selective outcome reporting and allocation concealment were deemed low in 12 and 11 studies, respectively. Six studies were categorized as low risk for incomplete outcome data and other sources of biases. Furthermore, high risk was noted in one study for blinding and in another for other sources of biases. Based on the quality grading criteria, six studies were rated as Grade A, four as Grade B, and three as Grade C.

### 3.4 Meta-analysis results

#### 3.4.1 Incidence of vertebral fractures

**Figure 2:**
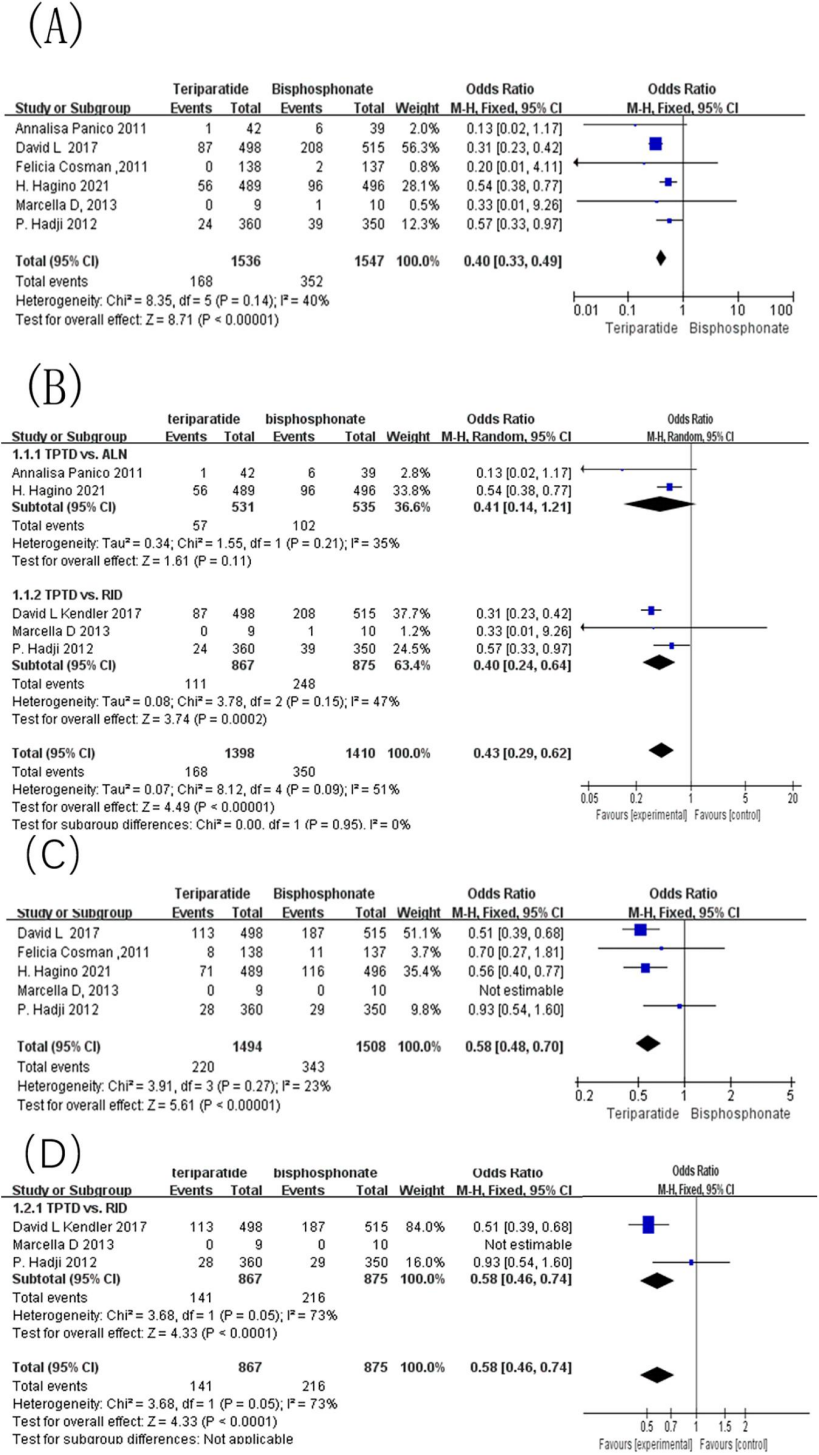
Forest map of fracture occurrence. **(A)** Forest map of the incidence of vertebral fracture incidence; **(B)** Forest map of the sensitivity analysis for the incidence of vertebral fractures; **(C)** Forest map of the incidence of non-vertebral fractures; **(D)** Forest map of the sensitivity analysis for the incidence of non-vertebral fractures.

Six studies included in the meta-analysis reported the incidence of vertebral fractures in patients with OP treated with either teriparatide or bisphosphonates. Of these, two studies used alendronate as the control, three used risedronate, and one used zoledronic acid. One study focused on male participants, whereas the remaining five studies included only female participants. As shown in Figure 2A, the combined effect sizes revealed an I^2^ of 40% and a *p*-value of 0.14, indicating low statistical heterogeneity among the studies. A fixed-effects model was consequently applied, revealing an OR of 0.40, 95% CI of [0.33, 0.49], and *p* < 0.00001.

After stratifying studies according to the type of bisphosphonate used in the control group, the result is shown in Figure 2B. When compared with alendronate, teriparatide showed a trend toward reducing vertebral fracture risk. However, the pooled effect did not reach statistical significance [OR = 0.41, 95% CI: (0.14, 1.21), *p* = 0.11], and heterogeneity remained low (I^2^ = 35%). In contrast, for trials using risedronate as the comparator, teriparatide demonstrated a significant reduction in vertebral fractures [OR = 0.40, 95% CI: (0.24, 0.64), *p* = 0.0002], accompanied by higher heterogeneity (I^2^ = 47%), suggesting that part of the variability may be attributed to differences in study design, follow-up duration, and baseline fracture risk among participants. Only one study used zoledronic acid as the comparator. Therefore, a pooled estimate could not be calculated for this agent. Importantly, the subgroup difference test did not show statistical significance (Chi^2^ = 0.00, df = 1, *p* = 0.95), indicating that although the magnitude of the effects varied slightly among different control types, the overall direction of the effects of alendronate and risedronate control trials was consistent. This suggests that the superior efficacy of teriparatide in preventing vertebral fractures appears to be maintained regardless of the specific bisphosphonate used as a comparator.

#### 3.4.2 Incidence of non-vertebral fractures

Five of the included studies reported the impact of teriparatide vs. bisphosphonates on the incidence of non-vertebral fractures in patients with OP. Among these, one study used alendronate as the control, three used risedronate, and one used zoledronic acid. One study focused on male participants, while the remaining four studies included only female participants. As shown in Figure 2C, the combined effect sizes revealed an I^2^ of 23% and a *p*-value of 0.27, indicating acceptable statistical heterogeneity among the studies. A fixed-effects model was consequently applied, revealing an OR of 0.58, 95% CI of [0.54, 0.70], and *p* < 0.00001. This result suggests that teriparatide is significantly more effective than bisphosphonates in reducing the incidence of non-vertebral fractures in patients with OP.

In order to further explore the potential influence of different bisphosphonate comparators on the non-vertebral fracture outcome, a subgroup analysis was conducted based on the type of control drug used. Because only one study used alendronate and one used zoledronate, pooled estimates could not be generated for these agents. A pooled analysis was feasible only for studies using risedronate as the comparator. As shown in Figure 2D, three studies were included in this subgroup. The combined effect size did not reach statistical significance [OR = 0.74, 95% CI: (0.49, 1.13), *p* = 0.16], and heterogeneity within this subgroup was high (I^2^ = 73%, *p* = 0.05). These results indicate that, for non-vertebral fractures, the relative advantage of teriparatide over risedronate may be less pronounced than that observed for vertebral fractures, and substantial variability remains among the available trials.

#### 3.4.3 12-month increase rate of lumbar spine BMD

**Figure 3:**
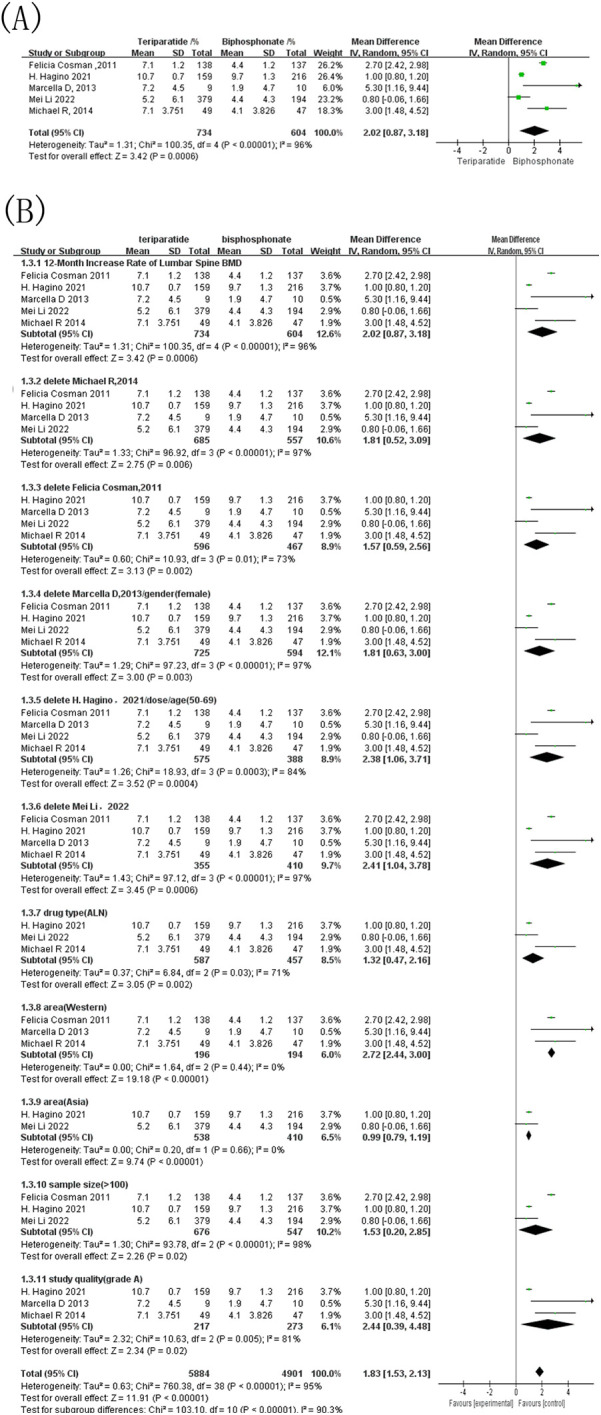
Forest map comparing the growth rate of lumbar bone density within 12 months after treatment. **(A)** Forest map of the 12-month increase rate of lumbar spine BMD; **(B)** Forest map of the sensitivity analysis for the 12-month increase rate of lumbar spine BMD.

Five studies included in the meta-analysis reported 12-month changes in the lumbar spine BMD in patients with OP treated with either teriparatide or bisphosphonates. Of these, three studies used alendronate as the control, while the remaining two studies used risedronate and zoledronic acid. As depicted in Figure 3A, the combined effect sizes revealed an I^2^ of 96% and a *p*-value < 0.00001, indicating substantial heterogeneity among the studies. Therefore, a random-effects model was applied, yielding a combined MD of 2.02 and a 95% CI of [0.87, 3.18]. This finding suggests that teriparatide significantly outperformed bisphosphonates in improving lumbar spine BMD in patients with OP (*p* < 0.00001).

Given the high heterogeneity, sensitivity analysis performed by sequentially omitting each study showed that the direction of the pooled effect and the overall heterogeneity remained largely unchanged, indicating that no single study accounted for the substantial heterogeneity observed in the primary analysis (Figure 3B). To further investigate potential sources of heterogeneity, we conducted a series of predefined subgroup analyses (Figure 3B) based on bisphosphonate type (alendronate vs. risedronate vs. zoledronic acid), sex (female vs. male), sample size (<100 vs. ≥ 100), participant age (50–69 vs. 70–89 years), geographic region (Western vs. Asian), and study quality (grade A). Among these strata, geographic region emerged as the most prominent modifier of heterogeneity. Western studies demonstrated a robust and highly consistent treatment effect (MD 2.72; I^2^ = 0%), whereas Asian studies showed a markedly attenuated effect (MD 0.99) with complete homogeneity (I^2^ = 0%). This striking contrast suggests that regional differences—potentially reflecting population characteristics, clinical practice patterns, or medication adherence—represent a key driver of heterogeneity in the overall analysis. Other subgroup factors, including drug type and study quality, contributed partially to the variability, yet heterogeneity within these subgroups generally remained above 50%. Additional subgroup divisions failed to produce any meaningful reduction in heterogeneity.

#### 3.4.4 12-month increase rate of femoral neck BMD

**Figure 4:**
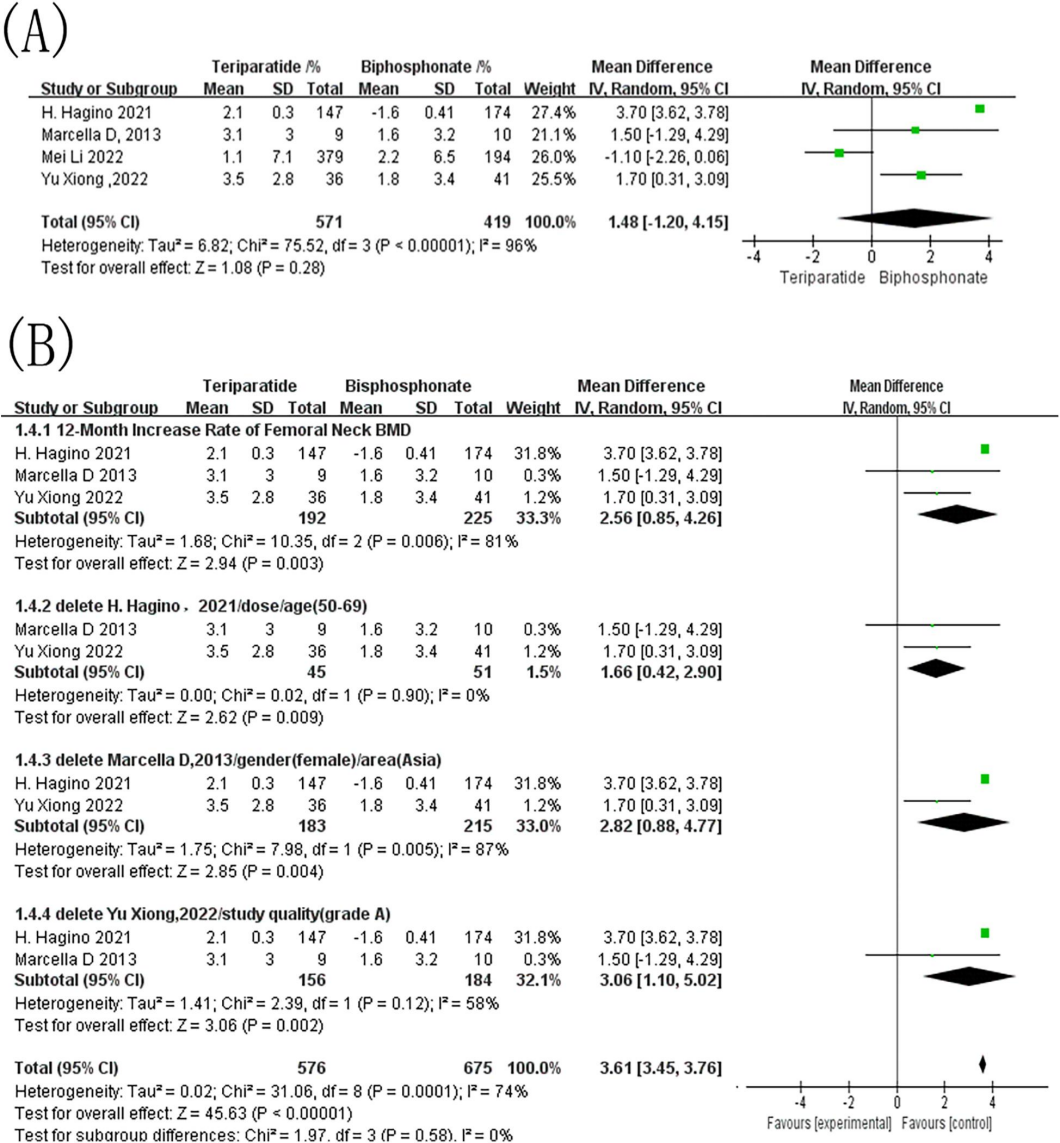
Forest map of bone mineral density growth rate of femoral neck within 12 months after treatment. **(A)** Forest map of the 12-month increase rate of femoral neck BMD; **(B)** Forest map of the sensitivity analysis for the 12-month increase rate of femoral neck BMD.

Three studies reported 12-month changes in femoral neck BMD in patients with OP treated with either teriparatide or bisphosphonates. The control drugs included alendronate, risedronate, and zoledronic acid. As shown in Figure 4A, the combined effect sizes revealed an I^2^ value of 81% and a *p*-value of 0.006, indicating significant heterogeneity among the studies. A random-effects model was therefore used, resulting in an MD of 2.56, 95% CI = [0.85, 4.26], and a *p*-value = 0.003. This result demonstrates that teriparatide significantly outperformed bisphosphonates in improving femoral neck BMD after 12 months. Given the potential impact of substantial heterogeneity on the results of the meta-analysis, sensitivity analysis was conducted (Figure 4B).

Sensitivity analyses based on sequential study exclusion revealed that the overall direction of the treatment effect remained unchanged across all scenarios, consistently favoring teriparatide. When the study by Hagino et al. was excluded (subgroup based on dose/age), heterogeneity dropped markedly from 81% to 0%, and the pooled estimate (MD = 1.66, 95% CI 0.42–2.90) remained significant. This suggests that differences related to baseline age distribution or dosing regimen may partially contribute to between-study variability. Additionally, subgrouping by study quality (removal of Yu Xiong et al.) only modestly reduced heterogeneity (I^2^ decreased from 81% to 58%), suggesting that methodological quality alone is unlikely to be the primary driver of heterogeneity. In contrast, subgrouping based on gender or region has little impact on heterogeneity.

#### 3.4.5 18-month or longer increase rate of lumbar spine BMD

**Figure 5:**
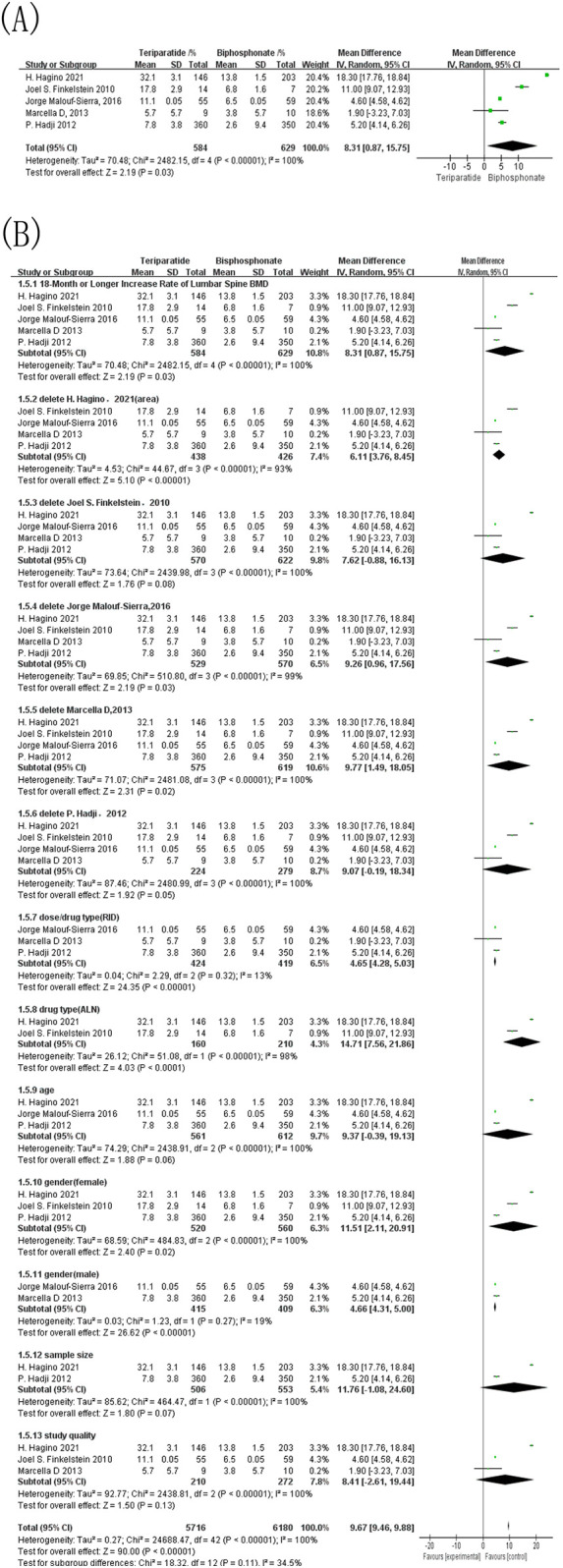
Forest map comparing the growth rate of lumbar bone density over 18 months of follow-up. **(A)** Forest map of the 18-month or longer increase rate of lumbar spine BMD; **(B)** Forest map of the sensitivity analysis for the 18-month or longer increase rate of lumbar spine BMD.

Five studies reported ≥18-month changes in lumbar spine BMD. As shown in Figure 5A, the pooled analysis favored teriparatide over bisphosphonates (MD = 8.31, 95% CI: 0.87–15.75; *p* = 0.03), but with extremely high heterogeneity (I^2^ = 100%). To further explore potential sources of variability, predefined subgroup analyses were performed (Figure 5B). When restricting the analysis to studies using risedronate as the comparator, heterogeneity dropped dramatically from 100% to 13%, and the treatment effect remained stable (MD = 4.65, 95% CI: 4.28–5.03; *p* < 0.00001). This suggests that mixing different bisphosphonates in the control group was a major contributor to the overall heterogeneity. Other subgroup analyses did not meaningfully reduce heterogeneity, which remained above 90%, suggesting that heterogeneity stems from multiple interacting sources.

#### 3.4.6 18-month or longer increase rate of femoral neck BMD

**Figure 6:**
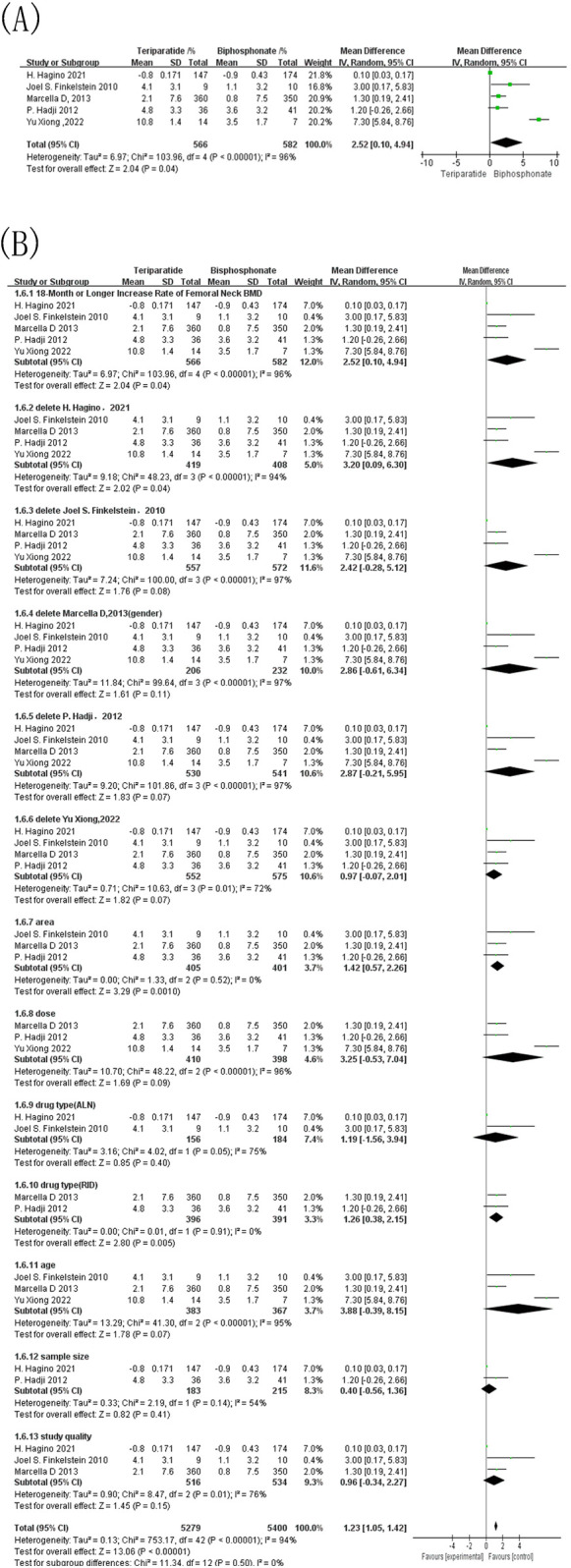
Forest map of bone mineral density growth rate of femoral neck over 18 months of follow-up. **(A)** Forest map of the 18-month or longer increase rate of femoral neck BMD; **(B)** Forest map of the sensitivity analysis for the 18-month or longer increase rate of femoral neck BMD.

Five studies included in this meta-analysis reported over 18 months of changes in femoral neck BMD in patients with OP under two different interventions. The control drugs used included alendronate, risedronate, and zoledronic acid. As shown in Figure 6A, the combined effect sizes revealed an I^2^ of 96% and a *p*-value of < 0.00001, indicating substantial statistical heterogeneity. A random-effects model was therefore used, yielding an MD of 2.52, 95% CI = [0.10, 4.94], and *p* = 0.04. This finding suggests that within 24 months after intervention, the bisphosphonate group exhibited a significantly lower average increase in femoral neck BMD compared to the teriparatide group. Given the substantial heterogeneity among the studies, sensitivity analysis was carried out (Figure 6B). Sensitivity and subgroup analyses revealed that heterogeneity dropped to 0% when studies were stratified by geographic region (Western) and bisphosphonate type (risedronate), whereas other subgroup factors had minimal impact. These findings indicate that regional differences and comparator drug type were the primary contributors to the observed heterogeneity.

#### 3.4.7 Adverse events

**Figure 7:**
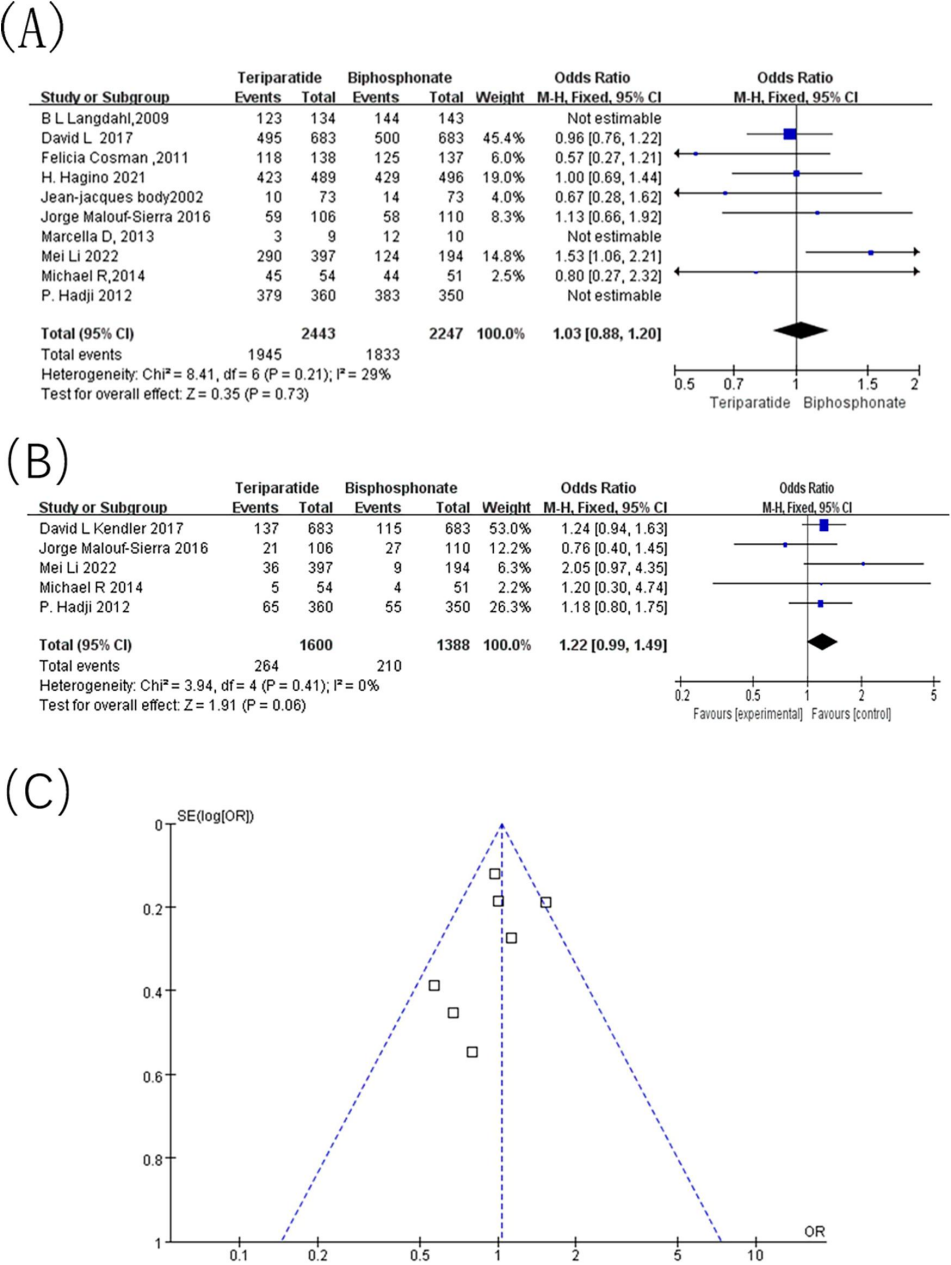
Adverse events. **(A)** Forest map of adverse events; **(B)** Forest map of serious adverse events; **(C)** Inverted funnel diagram of adverse event statistics.

A total of 10 studies reported adverse events associated with different treatment regimens. As depicted in Figure 7A, combined effect size analysis revealed a I^2^ of 29% and *p* value of 0.21, indicating low statistical heterogeneity. A fixed-effects model was subsequently employed, yielding an OR of 1.03, 95% CI = [0.88, 1.20], and *p* = 0.73, with no statistically significant difference. The meta-analysis suggests that there was no significant difference in the incidence of adverse events between teriparatide and bisphosphonates in treating OP.

To assess whether teriparatide was associated with a higher risk of severe or drug-specific adverse events, we compared the incidence of serious adverse events between the teriparatide and bisphosphonate groups (Figure 7B). Five studies (6, 10, 14, 15, 18) reported serious adverse events (SAEs). Pooled analysis showed no statistically significant difference between the two treatments [OR = 1.22, 95% CI = (0.99, 1.49), and *p* = 0.06], indicating that teriparatide does not confer an increased risk of serious adverse events compared with bisphosphonates.

#### 3.4.8 Publication bias

Publication bias was assessed using adverse events as an example. As depicted in Figure 7C, the funnel plot indicates that while the data points are spread across both sides of the funnel, they are not perfectly balanced or symmetrical, suggesting a potential for publication bias.

## 4 Discussion

Osteoporotic fractures are associated with substantial morbidity, mortality, and economic burden, underscoring the importance of selecting the most effective therapeutic strategy (1, 2). In this meta-analysis including 13 RCTs and 4,420 participants, teriparatide consistently demonstrated superior efficacy over bisphosphonates in reducing vertebral and non-vertebral fracture risk and improving BMD at both the lumbar spine and femoral neck, regardless of whether treatment lasted 12 or 18 months. These findings reaffirm the anabolic advantage of teriparatide, which stimulates bone formation through osteoblast activation and differentiation, contrasting with the antiresorptive mechanism of bisphosphonates (19).

Although the direction of effect was highly consistent across all outcomes, the degree of heterogeneity varied substantially. For fracture outcomes, heterogeneity remained low (I^2^ < 50%) even before stratification, and subgroup analyses based on bisphosphonate type did not materially change the results, indicating that comparator drug differences were not major contributors for these endpoints. However, for BMD outcomes—particularly ≥18-month lumbar spine BMD—heterogeneity was extremely high (I^2^ = 100%). Subgroup analyses revealed that heterogeneity decreased markedly only when risedronate was assessed separately (I^2^ reduced to 13%), suggesting that drug type may influence long-term BMD changes. Geographic area also had a noticeable impact on heterogeneity, with Western-region trials generally showing more consistent results than Asian trials. Importantly, across most other subgroup analyses (sex, age, sample size, treatment duration, study quality), heterogeneity remained very high, indicating that variability across studies was multifactorial and only partly explained by the examined factors. These findings highlight that the overall heterogeneity is likely driven predominantly by regional differences and bisphosphonate type, and that in many clinical subgroups, heterogeneity remains difficult to reduce. Randomized controlled trials with longer follow-up and standardized protocols are needed to more accurately estimate long-term effect sizes in the future.

Despite the high heterogeneity in some BMD outcomes, the uniform direction of treatment effects reinforces the robustness of the qualitative conclusion that teriparatide is more effective than bisphosphonates. Nevertheless, the exact magnitude of benefit, especially for long-term BMD improvement, should be interpreted cautiously. Differences in study design, baseline patient characteristics, adherence, and follow-up duration may have contributed to the observed variability. Well-designed, standardized clinical trials with harmonized protocols are needed to provide more precise effect estimates for long-term outcomes.

Safety analyses showed similar overall and serious adverse event rates between teriparatide and bisphosphonates, with no statistically significant differences. However, most studies did not report the specific nature of serious adverse events (e.g., hypercalcemia, osteonecrosis of the jaw), limiting the ability to compare drug-specific safety profiles. Future trials should provide more granular reporting of adverse events to enhance clinical relevance.

The predominance of postmenopausal women among included participants limits the generalizability of findings to men. Given physiological and hormonal differences in bone remodeling, dedicated trials in male osteoporosis are warranted. Additionally, treatment cost remains an important but underreported factor. While teriparatide demonstrates superior efficacy, its higher cost may affect real-world use, especially in resource-limited settings. Economic evaluations are needed to determine optimal treatment strategies across different healthcare systems. In line with the current guidelines of Bone Health and Osteoporosis Foundation, teriparatide is primarily indicated for patients at very high fracture risk, such as those with multiple spine fractures/hip fracture and T-score of−2.5 or lower at lumbar spine or hip. For patients with moderate risk, bisphosphonates remain the preferred first-line option. Sequential or combination strategies, where teriparatide is followed by an antiresorptive agent, may optimize both efficacy and cost-effectiveness (20).

This study has several limitations. Some outcomes were informed by a small number of RCTs, resulting in limited statistical power for certain subgroups. Important clinical factors—such as bone turnover markers, cost-related variables, and specific adverse effects—were not fully assessed. Heterogeneity in study populations, treatment durations, and bisphosphonate types may also affect the consistency of findings. Nonetheless, after rigorous screening, data extraction, and quality assessment, the included trials demonstrated high consistency in key outcomes and effect directions, lending credibility to the robustness of our meta-analytic conclusions.

Overall, this study provides a comprehensive synthesis of current evidence and confirms the superior efficacy of teriparatide over bisphosphonates in improving BMD and reducing fracture risk, with comparable safety profiles. These findings may help guide individualized treatment decisions and highlight areas requiring further well-designed clinical research.
